# The complete chloroplast genome sequence of medicinal plant: *Cynanchum thesioides* (Asclepiadaceae)

**DOI:** 10.1080/23802359.2021.1961622

**Published:** 2021-08-11

**Authors:** Peng Kang, Yuqian Guo, Yiyue Zhang, Yuqing Wei

**Affiliations:** Key Laboratory of Ecological Protection of Agro-pastoral Ecotones in the Yellow River Basin of National Ethnic Affairs Commission of the People’s Republic of China, College of Biological Science & Engineering, North Minzu University, Yinchuan, Ningxia, P. R. China

**Keywords:** Chloroplast genome, phylogenetic analysis, Asclepiadaceae, *Cynanchum thesioides*

## Abstract

*Cynanchum thesioides* is a medicinal plant. The complete chloroplast genome sequence of is 158,547 bp in length, contains 131 complete genes, including 85 protein-coding genes (85 PCGs), 8 ribosomal RNA genes (8 rRNAs), and 37 tRNA genes (37 tRNAs). The overall AT content of cp DNA is 62.1%, the corresponding values of the LSC, SSC, and IR regions are 63.7, 67.7, and 56.5%. Phylogenetic tree shows that *C. thesioides* was identified as the most divergent among the sequenced species of *Cynanchum* used.

*Cynanchum* L. is a large genus in the subfamily Asclepiadoideae (Apocynaceae) with approximately 200 species and a wide distribution throughout the world (Liede and Täuber 2002; Endress 2018). *Cynanchum thesioides* (Freyn) K. Schum. (Engler and Anton 1895) belonging to genus *Cynanchum*, is an upright, xerophytic shrub. *Cynanchum thesioides* has high medicinal value and the plant's fresh juice has been used to treat condyloma acuminatum, plants are rich in rubber and fiber., which can be used as industrial raw materials. plant's fruit edible and seed wool can be used as filling material (Li et al. 1995; Zhang et al. 2020). However, the chloroplast genome of *C. thesioides* has not been reported. In this study, we assembled the complete chloroplast genome of *C. thesioides*, hoping to lay a foundation for further research.

Fresh leaves of *C. thesioides* were collected from Alxa Youqi (Alxa, Inner Mongolia, China; coordinates: 101°42′E, 39°11′N) and dried with silica gel. The voucher specimen was stored in Sichuan University Herbarium (zhanglei, 136083334@qq.com) with the accession number of QTPLJQCHNO0293043. Total genomic DNA was extracted with a modified CTAB method (Doyle and Doyle 1987) and a 350-bp library was constructed. This library was sequenced on the Illumina NovaSeq 6000 system with 150 bp paired-end reads. We obtained 10 million high quality pair-end reads for *C. thesioides*, and after removing the adapters, the remaining reads were used to assemble the complete chloroplast genome by NOVOPlasty (Dierckxsens et al. 2017). The complete chloroplasts genome sequence of *C. wilfordii* was used as a reference. Plann v1.1 (Huang and Cronk 2015) and Geneious v11.0.3 (Kearse et al. 2012) were used to annotate the chloroplasts genome and correct the annotation.

The total plastome length of *C. thesioides* (MW864598) is 158,547 bp, exhibits a typical quadripartite structural organization, consisting of a large single copy (LSC) region of 90,848 bp, two inverted repeat (IR) regions of 24,321 bp, and a small single copy (SSC) region of 19,057 bp. The cp genome contains 131 complete genes, including 85 protein-coding genes (85 PCGs), 8 ribosomal RNA genes (8 rRNAs), and 37 tRNA genes (37 tRNAs). Most genes occur in a single copy, while 16 genes occur in double, including all rRNAs (4.5S, 5S, 16S, and 23S rRNA), 7 tRNAs (*trnA-UGC*, *trnI-CAU*, *trnI-GAU*, *trnL-CAA*, *trnN-GUU*, *trnR-ACG*, and *trnV-GAC*), and 5 PCGs (*rps7*, *ndhB*, *ycf2*, *rpl2*, *rpl23*). The AT content of the whole plastome is 62.1%, while those of the LSC, SSC, and IR regions are 63.7, 67.7, and 56.5%, respectively.

In order to further clarify the phylogenetic position of *C. thesioides*, plastomes of four representative of *Cynanchum* species were obtained from NCBI to reconstruct the plastome phylogeny, with *Calotropis procera* as an outgroup. All the sequences were aligned using MAFFT v.7.313 (Katoh and Standley 2013) and maximum likelihood phylogenetic analyses were conducted using RAxML v.8.2.11 (Stamatakis 2014) under GTRCAT model with 500 bootstrap replicates (Figure 1) . The phylogenetic tree shows that the species of *Cynanchum* were clustered together while *C. thesioides* was identified as the most divergent among the sequenced species of *Cynanchum* used.

**Figure 1.**
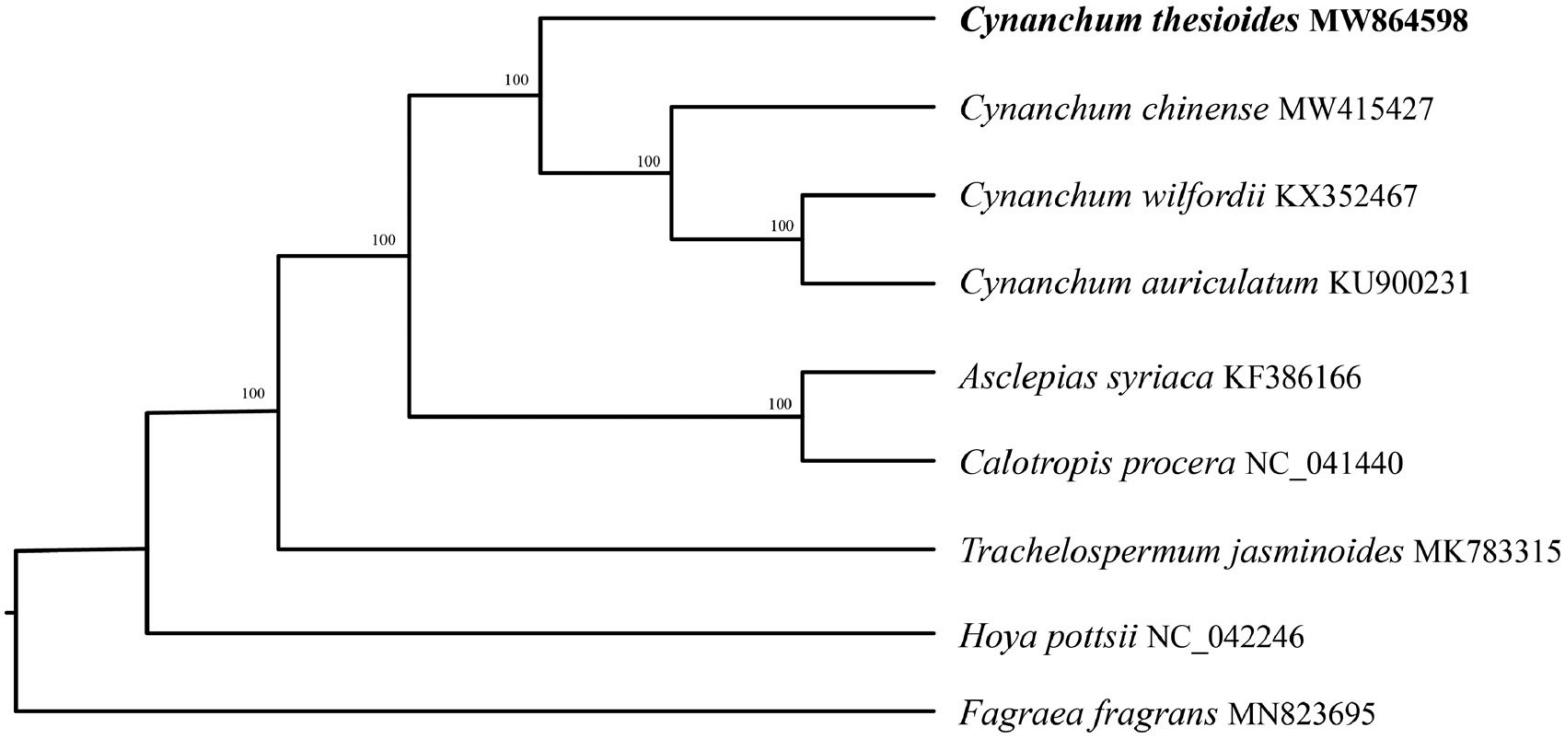
Phylogenetic relationships of *Cynanchum* species using whole chloroplast genome. GenBank accession numbers: *Asclepias syriaca* (KF386166)*, Calotropis procera* (NC_041440)*, Cynanchum auriculatum* (KU900231)*, Cynanchum chinense* (MW415427)*, Cynanchum thesioides* (MW864598)*, Cynanchum wilfordii* (KX352467)*, Fagraea fragrans* (MN823695)*, Hoya pottsii* (NC_042246)*, Trachelospermum jasminoides* (MK783315).

## Data Availability

The data that support the findings of this study are openly available in GenBank of NCBI at https://www.ncbi.nlm.nih.gov, reference number MW864598. The associated BioProject, SRA, and Bio-Sample numbers are PRJNA672277, SRA: SRS8756897, and SAMN18837310, respectively.
